# Post-Stroke Epilepsy in Young Adults: A Long-Term Follow-Up Study

**DOI:** 10.1371/journal.pone.0055498

**Published:** 2013-02-04

**Authors:** Renate Arntz, Loes Rutten-Jacobs, Noortje Maaijwee, Hennie Schoonderwaldt, Lucille Dorresteijn, Ewoud van Dijk, Frank-Erik de Leeuw

**Affiliations:** 1 Department of Neurology, Radboud University Nijmegen Medical Centre, Donders Institute for Brain, Cognition and Behaviour, The Netherlands; 2 Department of Neurology, Medisch Spectrum Twente, The Netherlands; University of South Florida, United States of America

## Abstract

**Background:**

Little is known about the incidence and risk of seizures after stroke in young adults. Especially in the young seizures might dramatically influence prognosis and quality of life. We therefore investigated the long-term incidence and risk of post-stroke epilepsy in young adults with a transient ischemic attack (TIA), ischemic stroke (IS) or intracerebral hemorrhage (ICH).

**Methods and Findings:**

We performed a prospective cohort study among 697 consecutive patients with a first-ever TIA, IS or ICH, aged 18–50 years, admitted to our hospital between 1-1-1980 till 1-11-2010. The occurrence of epilepsy was assessed by standardized questionnaires and verified by a neurologist. Cumulative risks were estimated with Kaplan-Meier analysis. Cox proportional hazard models were used to calculate relative risks. After mean follow-up of 9.1 years (SD 8.2), 79 (11.3%) patients developed post-stroke epilepsy and 39 patients (5.6%) developed epilepsy with recurrent seizures. Patients with an initial late seizure more often developed recurrent seizures than patients with an initial early seizure. Cumulative risk of epilepsy was 31%, 16% and 5% for patients with an ICH, IS and TIA respectively (Logrank test ICH and IS versus TIA p<0.001). Cumulative risk of epilepsy with recurrent seizures was 23%, 8% and 4% respectively (Logrank ICH versus IS p = 0.05, ICH versus TIA p<0.001, IS versus TIA p = 0.01). In addition a high NIHSS was a significant predictor of both epilepsy and epilepsy with recurrent seizures (HR 1.07, 95% CI 1.03–1.11 and 1.08, 95% CI 1.02–1.14).

**Conclusions:**

Post-stroke epilepsy is much more common than previously thought. Especially patients with an ICH and a high NIHSS are at high risk. This calls upon the question whether a subgroup could be identified which benefits from the use of prophylactic antiepileptic medication. Future studies should be executed to investigate risk factors and the effect of post-stroke epilepsy on quality of life.

## Introduction

The incidence of epilepsy after stroke in elderly ranges from 2% to 14%, depending on study population, stroke subtype and definitions of epilepsy [1]–[7]. Post-stroke epilepsy has a negative effect on stroke recovery and quality of life [7]–[10]. However information on post-stroke epilepsy is only available from studies containing elderly stroke patients, which can not necessarily be generalized to the young. Especially in young patients with a long life expectancy, seizures might have a dramatic influence on quality of life and disability due to its negative effect on stroke recovery and unpredictability. Due to this unpredictability, work may be considered unsuitable or unsafe by employers, which could ultimately result in unemployment of these young patients. Thereby seizures may not only influence life of the individual patient, but may have an impact on family and community as well [11]. In addition the necessity to use long-term antiepileptic medication might have a particular high impact given their active lives with work, young families and many social activities. Surprisingly little is known about the long-term incidence and risk factors of post-stroke epilepsy after a stroke in young patients. The only studies performed so far were usually small, had a short follow-up and different definitions of post-stroke epilepsy. In addition those studies were not designed to investigate the risk of epilepsy and only included patients with an IS and not those with a TIA or ICH [3], [12]–[15].

Quantification of post-stroke epilepsy frequency and risk factors is a first step in both informing young stroke patients and evaluating and developing treatment strategies. The aim of this study was therefore to investigate the long-term incidence and risk of post-stroke epilepsy and epilepsy with recurrent seizures in patients with a TIA, IS or ICH at young age.

## Methods

### Patients and Study Design

This study is part of the “***F***ollow***-U***p of ***T***ransient ischemic attack and stroke patients and ***U***nelucidated ***R***isk factor ***E***valuation”-study (***FUTURE*** study), a prospective cohort study that investigates causes and consequences of stroke in young adults. Details of the study have been described elsewhere [16]. The Medical Review Ethics Committee region Arnhem-Nijmegen approved the study.

The FUTURE study comprises all consecutive patients with a TIA, IS or ICH, aged 18–50 years, admitted to the Radboud University Nijmegen Medical Centre from 1-1-1980 till 1-11-2010. TIA was defined as a rapidly evolving focal neurological deficit, without positive phenomena such as twitches, jerks or myoclonus, with no other than a vascular cause lasting less than 24 hours. Stroke was defined as focal neurological deficit persisting for more than 24 hours [17], [18]. On basis of radiological findings, stroke was subdivided into ICH and IS. Exclusion criteria were previous stroke or TIA, traumatic hemorrhagic stroke, hemorrhage in known cerebral metastasis or primary brain tumor, cerebral venous sinus thrombosis, subarachnoid hemorrhage or ICH due to known ruptured aneurysm and retinal infarction. For the present study only patients with a first-ever stroke were included and history of seizures was an additional exclusion criteria.

Patients were included prospectively between 1980 and 2010 with a standardized collection of baseline and clinical characteristics (including demographics, stroke subtype, vascular risk factors and a history of epilepsy). Furthermore all patients underwent neurological examination and brain imaging at the time of their index event [16]. The assessment of stroke etiology (TOAST [19]) and severity (National Institutes of Health Stroke Scale (NIHSS) [20] and modified Rankin scale [21]) was done for all cases retrospectively by a validated approach, [22], [23] as these scales did not exist at the time when a substantial proportion of our patients experienced their qualifying event.

### Follow-up

Information on the vital status was available either from the hospital or via the municipality registry. Patients alive were invited for follow-up assessment between 1-11-2009 and 1-1-2012. All patients signed informed consent.

### Post-stroke Epilepsy

Primary outcome was the occurrence of epilepsy after stroke. Post-stroke epilepsy was defined according to the most recent version of the International League Against Epilepsy (ILAE), in which patients with a single seizure associated with an enduring condition that could cause epilepsy (for example stroke), met the criteria of epilepsy [24]. Seizures were classified as partial (simple, complex and secondary generalized) or generalized [25]. If it was not possible to classify a seizure, this was defined as not otherwise specified. Furthermore seizures were classified according to their time of onset. Seizures occurring within 1 week after stroke were classified as early seizures and seizures occurring thereafter were classified as late seizures [26]. Onset seizures were defined as occurring within 24 hours of onset of stroke [2]. To provide comparability with other studies epilepsy with recurrent seizures was included as a secondary outcome.

All patients alive underwent standardized, structured questionnaires. In case a patient had died, this information was retrieved from the general practitioner by the same structured questionnaires. If seizures were reported, information from the treating physician was retrieved, both for patients alive and patients who died. This information was verified and adjudicated by an experienced neurologist who was blinded for the index event. In addition when patients reported early or onset seizures, this information was verified by medical chart review or by retrieving information from other treating physicians. Information about date of seizure, number of seizures during follow-up and treatment were systematically recorded. Data on outcome were available for all patients.

### Statistical Analysis

For comparison of continuous variables between groups students-t-test or Mann-Whitney U test were used when appropriate. In case of categorical variables chi-square-test was used.

Cumulative risks of post-stroke epilepsy and epilepsy with recurrent seizures were estimated with Kaplan-Meier analysis, with the first post-stroke seizure as qualifying endpoint. Patients who died or did not reach the endpoint before follow-up ended were censored. Furthermore as patients with a recurrent stroke or TIA might have a higher risk of epilepsy, they were censored after their recurrent event. Differences between stroke subtype and gender in cumulative risks of post-stroke epilepsy and epilepsy with recurrent seizures were tested with the log rank test.

Average annual risks of epilepsy and epilepsy with recurrent seizures were calculated using the formula 1−[(1−I_c_)^1/n^] where I_c_ equals the cumulative risk of epilepsy or epilepsy with recurrent seizures at n years, obtained by the Kaplan-Meier method [27]. Cox proportional hazard models were used to calculate hazard ratios with their corresponding 95% confidence intervals for gender, age at event, stroke subtype and NIHSS at admission.

Two-sided P values of less than 0.05 were considered to indicate statistical significance. SPSS 18 was used for all statistical analysis.

## Results

### Study Population

**Figure 1:**
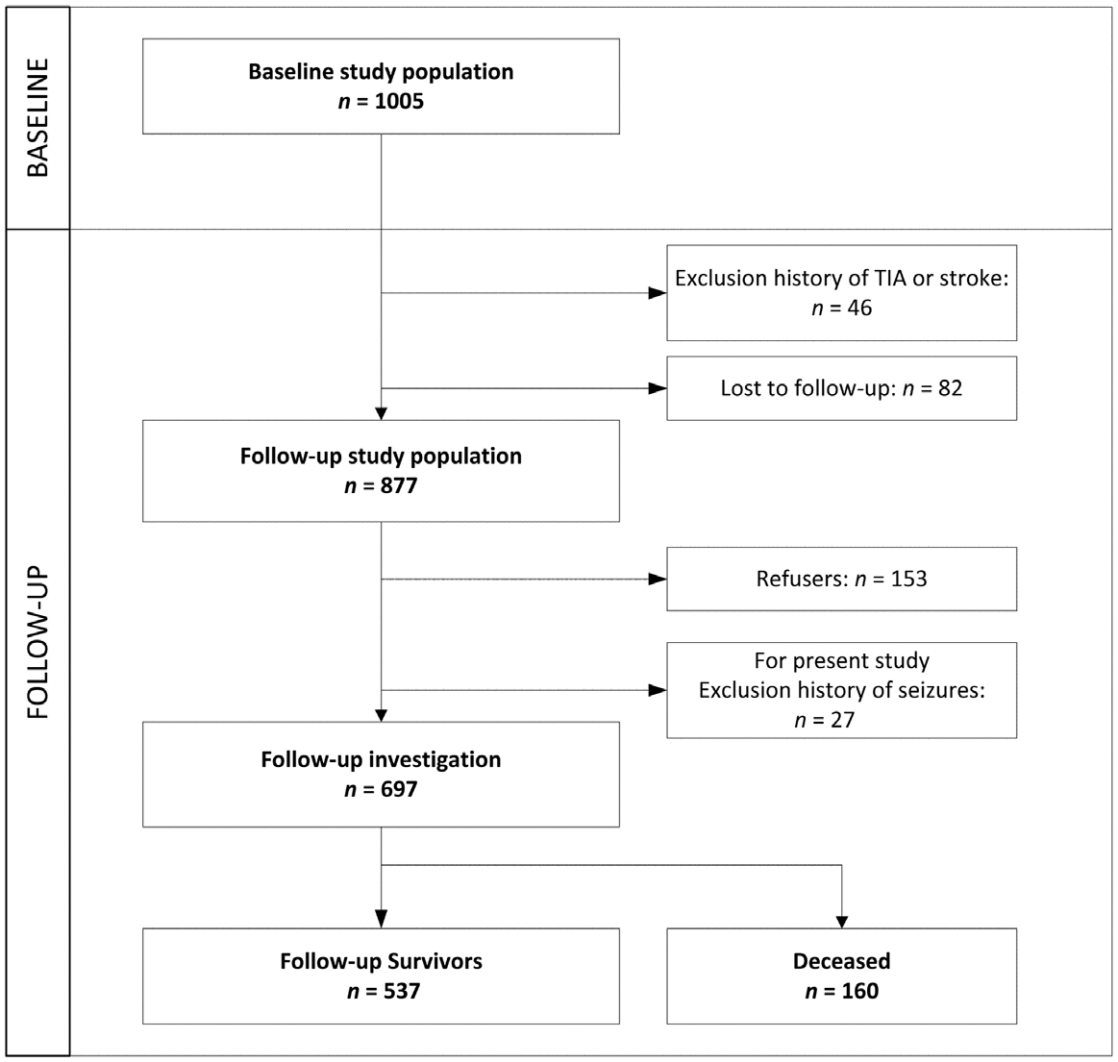
Flowchart study design.

1006 patients fulfilled in- and exclusion criteria of the FUTURE study, after inclusion one more patient with an ICH was excluded because after careful reevaluation this appeared to be on the basis of a cerebral venous sinus thrombosis, which was an exclusion criteria for our study. For the present study 697 patients were included in the analysis (Figure 1). Our study included 206 patients with a TIA (29.6%), 425 patients with an IS (61.0%) and 66 patients with an ICH (9.5%). Compared to participants, patients lost to follow-up more often had an IS (76.5% versus 61.0%, p = 0.008) and less often a TIA (13.1% versus 29.0%, p = 0.002). Furthermore patients lost to follow-up were younger (38.1 versus 40.5 years, p = 0.02) and less often had diabetes (2.5% versus 5.7%, p = 0.006). Except for smoking there were no differences in baseline characteristics in patients who refused to participate and participants (53.1% versus 62.0%, p = 0.05).

Participants’ mean age was 40.5 years (SD 7.8) and 47.1% was male. Median NIHSS at admission was 3 (IQR 1–8.5). Of all patients 160 (23.0%) had died after a mean follow-up of 9.1 years (SD 8.2).

### Frequency of Post-stroke Epilepsy

79 patients (11.3%) developed post-stroke epilepsy during follow-up (Table 1). In 25 patients the first seizure was an early seizure and thus occurred within 1 week after stroke. In 53 patients the first seizure was a late seizure. Patients with an ICH had the highest incidence of epilepsy (16.7%), followed by IS (14.4%) and patients with a TIA (3.4%). Classification of the first seizure is shown in table 2.

**Table 1:** Baseline Characteristics.

		Post-stroke epilepsy		
	Total, n = 697	Absent, n = 618 (%)	Present, n = 79 (%)	*p-*value
stroke subtype				
TIA	206 (29.6)	199 (32.2)	7 (8.9)	
IS	425 (61.0)	364 (58.9)	61 (77.2)	<0.001*
ICH	66 (9.5)	55 (8.9)	11 (13.9)	<0.001*
Mean age (SD)	40.5 (7.8)	40,7 (7.9)	39.2 (7.4)	0.03
Sex, male	369 (47.1)	334 (54.0)	35 (44.3)	0.10
Mean follow-up (SD)	9.1 (8.2)	9.0 (8.2)	10.5 (8.4)	0.09
NIHSS, median (IQR)‡	3 (1–8.5)	3 (1–8)	8.5 (2–14)	<0.001
TOAST§				
Large artery	161(25.5)	146 (25.9)	15 (22.1)	0.50
Cardio-embolism	84 (13.3)	69 (12.3)	15 (22.1)	0.03
Lacunar	60 (9.5)	60 (10.7)	0	0.005
Other determined	91 (14.4)	72 (12.8)	19 (27.9)	<0.001
Multiple	17 (2.7)	15 (2.7)	2 (29)	0.89
Undetermined	218 (34.5)	201 (35.7)	17 (25.0)	0.08
Cardiovascular risk factors				
DM	40 (5.7)	38 (6.1)	2 (2.5)	0.19
Hypertension	145 (20.8)	136 (22.0)	9 (11.4)	0.03
Atrial fibrillation	12 (1.7)	11 (1.8)	1 (1.3)	0.74
Smoking‡	396 (61.8)	346 (54.8)	50 (69.4)	0.12

* Patients with ICH or IS compared to TIA both *p*<0.001. Patients with IS compared to ICH *p* = 0.62.

‡ In 0.6% of the patients NIHSS at admission and in 8% smoking status was missing.

§ Percentages TIA and IS patients.

Abbreviations: DM = diabetes mellitus.

**Table 2:** Classification of first post-stroke seizure.

Classification of seizures	Totaln = 76 (%)	TIAn = 7 (%)	ISn = 61 (%)	ICHn = 11 (%)
Partial				
Simple partial	25 (31.6)	3 (42.9)	19 (31.1)	3 (27.3)
Complex partial	4 (5.1)	1 (14.3)	3 (4.9)	0
Secondary generalized	21 (26.6)	2 (28.6)	19 (31.1)	4 (36.4)
Generalized				
Tonic-clonic	25 931.6)	1 (14.3)	16 (26.2)	4 (36.4)
Not otherwise specified	4 (5.1)	0	4 (6.4)	0

39 Patients (5.6%) had epilepsy with recurrent seizures during follow-up. Patients with an ICH more often developed epilepsy with recurrent seizures (10.6%), followed by IS (6.6%) and TIA patients (1.9%). Patients with an initial late seizure, more often developed recurrent seizures than patients with an initial early seizure (Table 3).

**Table 3:** Recurrence of seizures according to time of onset first seizure.

	Early seizures* (n = 25)			Late seizures‡ (n = 54)		
	No recurrence, n (%)		Recurrent seizures, n (%)	No recurrence, n (%)	Recurrent seizures, n (%)	*p*-value§
Overall	17 (68.0)		8 (32.0)	23 (42.6)	31 (57.4)	0.04
Men	10 (76.9)		3 (23.1)	12 (38.7)	19 (61.3)	0.02
Women	7 (58.3)		5 (41.7)	11 (47.8)	12 (52.2)	0.56
Stroke subtype						
TIA		1 (100.0)	0	3 (50.0)	3 (50.0)	0.35
IS		14 (70.0)	6 (30.0)	19 (46.3)	22 (53.7)	0.08
ICH		3 (75.0)	1 (25.0)	1 (14.3)	6 (85.7)	0.04

* early seizures are defined as occurring within the first week after the index event.

‡ late seizures are defined as occurring after the first week after the index event.

§ *p-*value represents difference in incidence of recurrent seizures between patients with an initial first seizure versus patients with an initial late seizure, compared with chi-square.

### Treatment

12 out of the 25 patients with an early seizures did not start anti-epileptic drugs (AED’s) during follow-up. 10 Patients started directly after the initial early seizure and 3 patients started after recurrent seizures. Of the 54 patients with an initial late seizure, 7 were not treated with AED’s during follow-up. 37 Started directly after the initial late seizure, 8 started after recurrent seizures and in 2 patients it was unknown when anti-epileptic drugs were started.

Patients with an initial late seizure more often started with AED’s than patients with an initial early seizures (87% versus 52%, p<0.001). Patients with recurrent seizures more often received AED’s than those with a single seizure (94.9% versus 57.5%, p<0.001).

### Risk of Post-stroke Epilepsy

**Figure 2:**
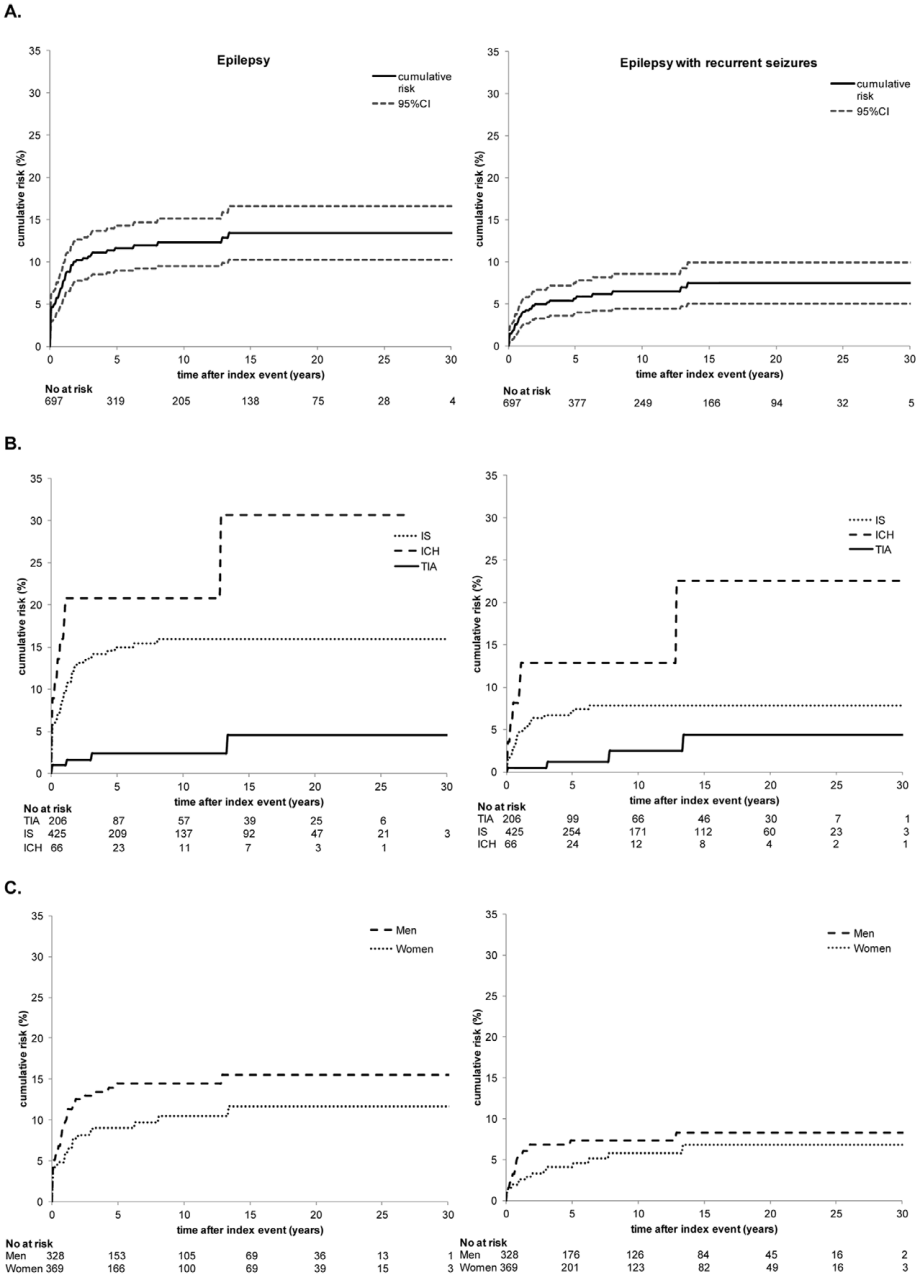
Average annual risk. **A.** Average annual risk of epilepsy. **B.** Average annual risk of epilepsy with recurrent seizures.

After total follow-up of maximum 30 years the cumulative risk of post-stroke epilepsy was 14% (95% CI 10.3–16.6) for all stroke subtypes. Cumulative risk of post-stroke epilepsy was 31%, 16% and 5% for patients with an ICH, IS and TIA respectively (log rank test ICH versus IS and IS versus TIA p<0.001) (Figure 2).

Cumulative risk of epilepsy with recurrent seizures was 7% (95% CI 5.0–10.0). Patients with an ICH (23%) or an IS (8%) had a higher cumulative risk of developing epilepsy with recurrent seizures than patients with a TIA (4%) (Log Rank ICH versus IS p = 0.051, ICH versus TIA p<0.001, IS versus TIA p = 0.013) (Figure 2).

**Figure 3:**
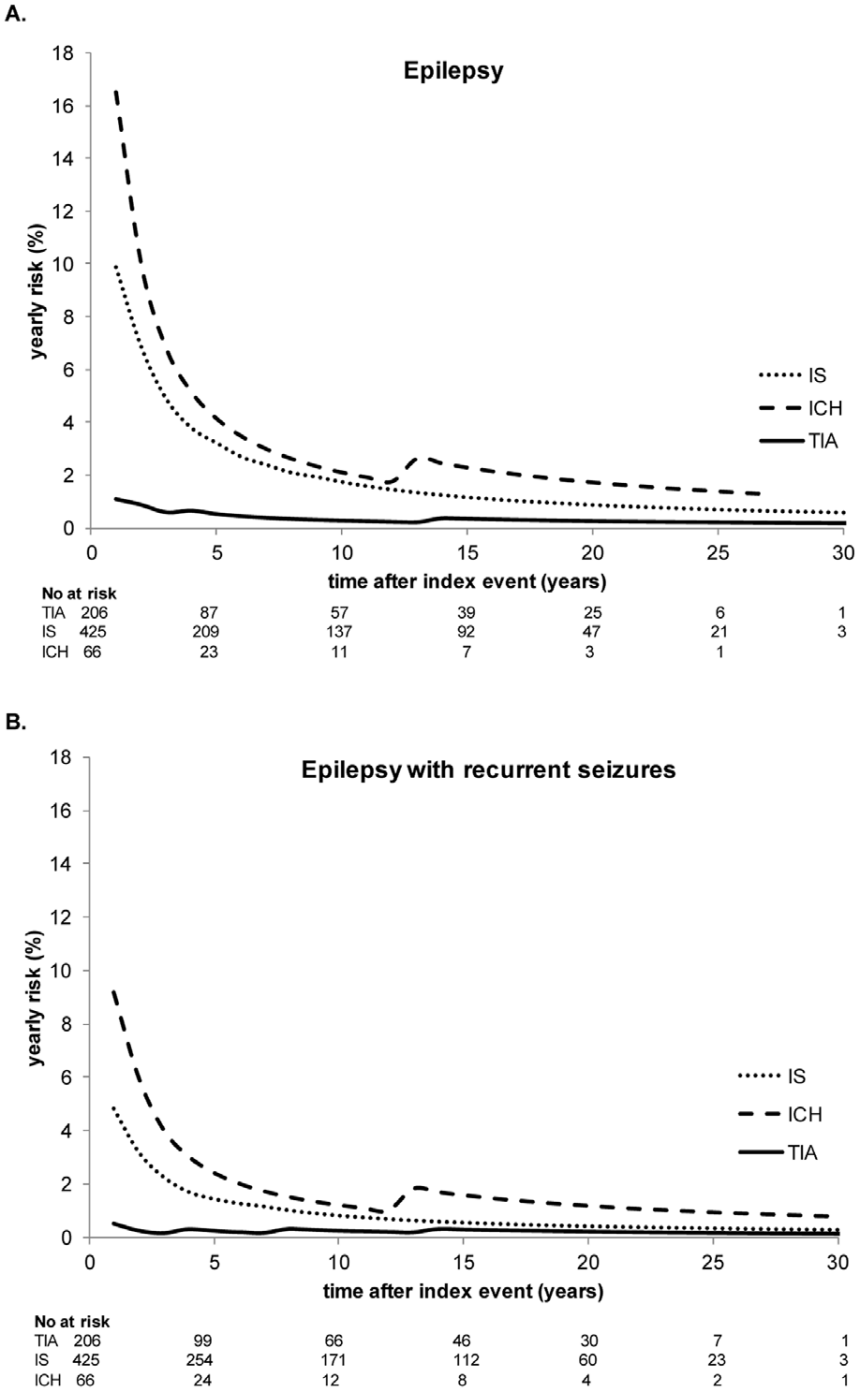
Cumulative risks for epilepsy and epilepsy with recurrent seizures. **A.** Overall cumulative risk. **B.** Cumulative risk according to stroke subtype. **C.** Cumulative risk according to gender.

Average annual risk of both epilepsy and epilepsy with recurrent seizures was the highest during the first year after the index event (8% and 4% respectively), especially in patients with an IS or ICH (Figure 3).

High NIHSS at admission and IS or ICH as stroke subtype were significant predictors of post-stroke epilepsy, corrected for age and gender (Table 4). High NIHSS remained a significant predictor for epilepsy with recurrent seizures.

**Table 4:** Cox proportional hazard.

	Epilepsy*		Epilepsy with recurrent seizures	
Kolom1	HR (95% CI)	*p*-value	HR (95% CI)	*p*-value
Stroke subtype				
IS vs TIA	3.68 (1.42–9.51)	0.007	2.23 (0.74–7.75)	0.16
ICH vs TIA	3.84 (1.20–12.28)	0.02	3.16 (0.79–12.67)	0.10
Male gender	0.72 (0.45–1.56)	0.18	1.28 (0.67–2.43)	0.46
Age at index event	0.98 (0.96–1.01)	0.23	0.98 (0.94–1.02)	0.24
NIHSS at admission	1.07 (1.03–1.11)	<0.001	1.08 (1.02–1.14)	0.005

* epilepsy is defined as the occurrence of at least 1 seizure with an enduring cause.

## Discussion

Our study is the first to investigate the incidence and risk of post-stroke epilepsy in young adults with a TIA, IS or ICH. We found an incidence of 11.3% for post-stroke epilepsy and 5.9% for epilepsy with recurrent seizures after a mean follow-up of almost 10 years.

Strengths of our study were the prospective and single centre design, which allowed us to collect information systematically and to uniformly verify both the qualifying event as well as the outcome. Furthermore, our study has one of the longest follow-up ever reported and the largest cohort compared with other studies investigating epilepsy after a stroke in young adults. In addition, our study has a high external validity, when comparing baseline characteristics to other studies [28].

However there are also a few limitations. Some selection bias may have occurred due to selective loss to follow-up. Those lost to follow-up more often had an IS and less often a TIA compared to participants. Potentially, this might have resulted in an underestimation of the overall risk of epilepsy in our cohort, as patients with an IS exhibited a higher risk of post stroke epilepsy. However, the severity of stroke did not differ between participants and those lost to follow-up, so we considered this loss of follow-up very unlikely to influence our results. Patients who refused to participate did not differ in baseline characteristic from participants except from smoking, making selection bias in this group for the same reason unlikely.

As information on seizures was collected by structured questionnaires for all patients in the same way, it is unlikely that seizures are reported differently between the stroke subtypes, which makes information bias very unlikely. Furthermore the neurologist verifying the event was blinded for the index event. Since the present study features a long inclusion period recall bias might have influenced our findings, especially when the index event or seizure occurred a longer time ago. However there were no differences in reporting seizures between patients who suffered their stroke a long time ago than those with a more recent stroke (9.0% in 2000–2010 versus 16.0% in 1990–1999 versus 11.6% in 1980–1989, p = 0.055), making recall bias unlikely. In addition it might have been possible that less information would be available in patients who died, especially in those who died a longer time ago, resulting in an underestimation of epilepsy in this group. However we did not find a difference in frequency of epilepsy between patients who died a long time ago and those who died more recently (12.5% in 1980–1989 versus 16.3% in 1990–1999 versus 16.7 in 2000–2010, p = 0.882), making this explanation unlikely. On top of this, information on patients who died before follow-up ended was collected in an identical manner as in patients alive.

Another source of bias might be misclassification of TIA patients. In some cases it might be difficult to distinguish a TIA from a seizure, especially for example in case of a limb-shaking TIA [29]. Theoretically either the index event or the seizure could have been misclassified. In our study all qualifying events were verified by two independent neurologists, independent from the outcome. In addition, 5 out of 8 patients developed complex partial seizures or (secondary) generalized seizures, which can be easily distinguished from TIA’s, so it is unlikely that those were misdiagnosed.

Another source of bias to be considered is confounding. We tried to overcome this by making appropriate adjustments for potential confounders including age, stroke severity, gender and stroke subtype in our multivariate analysis. Furthermore we have adjudicated for recurrent stroke or TIA, by withdrawing patients after a recurrent event in the Kaplan-Meier analysis.

Another possible confounding factor might have been the use of AED’s. In our study 10 out of 25 patients with an early seizure and 37 out of 54 patients with an initial late seizure started directly after the first seizure with AED’s. It might be possible that the use of AED’s has reduced the risk of epilepsy with recurrent seizures after a single seizures. However as AED’s were not prescribed prophylactic, only the frequency of epilepsy with recurrent seizures might have been affected by the use of AED’s and not the incidence of the first seizure. In addition the impact of AED’s on epileptogenesis remains unclear. Furthermore during 30 years of follow-up patients may have switched or stopped AED’s, which may have provoked or prevented occurrence of seizures as well.

According to the new definition of the ILAE patients already meet the criteria of epilepsy when a single seizure occurs with an enduring cause. Most other studies still use the former version in which patients meet the diagnosis of epilepsy when they have at least 2 (late) seizures. For reasons of comparability, we therefore compared the risks of seizures in previous studies with the risk of epilepsy in our study. We found a higher incidence of post-stroke epilepsy compared to other studies that investigated seizures stroke in young adults (6–10%) [3], [12]–[15]. This may be explained by the characteristics of our study. Except for IS and TIA patients we included patients with an ICH who had a higher cumulative risk of post-stroke epilepsy and epilepsy with recurrent seizures, whereas other studies only included ischemic strokes. For patients with an IS incidence of post-stroke epilepsy was almost 15% in our study, while others reported incidences of seizures between 6–10% [3], [12]–[15]. This difference may, in part, be explained by the far longer follow-up of our patients, leaving them more time to develop epilepsy. However a higher risk was found especially during the first years after the index event.

Compared to other studies in elderly with more or less the same definitions we found a higher incidence of epilepsy and epilepsy with recurrent seizures (3.4% to 10.4% and 2% to 5% respectively) [1], [2], [4], [6], [9]. Compared to the few studies in both young and elderly stroke patients which performed survival analysis, cumulative risk of post-stroke epilepsy and epilepsy with recurrent seizures was higher after same follow-up [2], [3], [6], [13].

The higher cumulative risk of post-stroke epilepsy and epilepsy with recurrent seizures in our young cohort may presumably be due to mechanisms in young patients which makes them more vulnerable for the development of epilepsy. This is also suggested by few other studies among elderly who found a higher incidence of seizures in younger patients, after stratification in several age groups [30]–[32]. It was suggested that the brain of elderly patients might have a weaker epileptogenicity [31], however there is no proper explanation for this finding yet.

Interestingly, in our study patients with a TIA had comparable risk of epilepsy than shown in studies among elderly TIA patients [1], [8], [32]–[34]. Despite the potential of misclassification of TIA patients, TIA was diagnosed according to current clinical guidelines and patients with positive phenomena such as twitches, jerks or myoclonus were excluded in order to minimize this, the only way to assess the risk of epilepsy after TIA is by including these patients in a study and ours is the first to do so.

Few studies found comparable incidences of early seizures, [1], [5]–[7], [35] however definitions of early seizures in those studies varied from 24 hours until 2 weeks, which obviously influences the incidence. In line with another study, we found that patients with initial late seizures more often developed epilepsy with recurrent seizures than those with initial early seizures [9]. One explanation might be the effect of survival, however there was no difference in survival between patients with an initial early and initial late seizure. In our opinion late seizures have a greater clinical importance, as those seizures very much influence daily life when patients are trying to rebuild their normal activities. In addition it has been suggested that pathophysiology of early and late seizures might be different. Early seizures might be related to non-cerebral disarrangements and thought to result from cellular biochemical dysfunction [4], [33], [36]. Late seizures on the other hand are suggested to be a result of gliosis or hemosiderin remaining [33].

As in other cohorts a higher cumulative risk epilepsy and epilepsy with recurrent seizures was found in patients with an ICH. In addition a high NIHSS was a significant predictor of both epilepsy and epilepsy with recurrent seizures [2], [9], [13], [30], [32], [37].

In conclusion, we showed that epilepsy and epilepsy with recurrent seizures are much more common after a stroke in young adults than previously described. Future studies should be executed to investigate risk factors and the underlying pathophysiology. Besides this, these studies should also investigate the effect of post-stroke epilepsy on quality of life. Furthermore, the high cumulative risk of epilepsy is as high as the risk of recurrent stroke [28]. This calls upon the question whether a subgroup could be identified, which would benefit from prophylactic AED’s, especially during the first years after stroke during which the risks are the highest.
